# An example of host plant expansion of host-specialized *Aphis gossypii* Glover in the field

**DOI:** 10.1371/journal.pone.0177981

**Published:** 2017-05-17

**Authors:** Dao-Wu Hu, Shuai Zhang, Jun-Yu Luo, Li-Min Lü, Jin-Jie Cui, Xiao Zhang

**Affiliations:** 1 State Key Laboratory of Cotton Biology, Institute of Cotton Research, Chinese Academy of Agricultural Sciences, Anyang, Henan, China; 2 Key Laboratory of Plant Stress Biology, College of Life Sciences, Henan University, Kaifeng, Henan, China; Helmholtz Zentrum Munchen Deutsches Forschungszentrum fur Umwelt und Gesundheit, GERMANY

## Abstract

The host plant expansion of host-specialized *Aphis gossypii* (Glover) has been well studied in the laboratory; however, this phenomenon is poorly understood in the field. Here, we provide a series of laboratory and field experiments to assess the role of zucchini in the host plant expansion of cotton-specialized aphids. We observed that cotton-specialized aphids possessed the ability to expand on a new host plant (cucumber), with individuals first recorded on June 12 and consequently increasing exponentially in number in a field cage. A bioassay experiment showed that aphids from both cotton and cucumber preferred their natal host, but clones from zucchini have a stronger preference for cucumber than cotton or zucchini. A total of 1512 individuals were collected from a cotton field (mixed cotton and cucurbit plot), cotton farmland (cotton alone) and a field cage and sequenced to identify their biotypes. The results for apterous individuals from the cotton field showed that more cucurbit-specialized biotypes occurred on cucumber and more cotton-specialized biotypes occurred on cotton and zucchini. A majority (> 97.0%) of aphids from both the field cage and cotton farmland were cotton-specialized individuals. Consequently, eliminating intermediate host plants may be an effective measure to suppress *A*. *gossypii* outbreaks, because cotton and cucumber are often grown together in fields and greenhouses.

## Introduction

The cotton—melon aphid, *Aphis gossypii* (Glover) (Hemiptera: Aphididae), is a notorious pest worldwide and has a large range of host plants including cotton, cucumber and zucchini [1]. *A*. *gossypii* can cause severe damage in commercial fields and urban green landscapes. The honeydew that aphid excretes decreases the photosynthetic activity of plants and contaminates fruit, resulting in severely reduced quality [2]. Moreover, *A*. *gossypii* can transmit more than 80 kinds of viral diseases that can cause substantially greater losses than the damage from direct feeding of the insect [3].

Populations of *A*. *gossypii* are differentiated by host plant preference, and previous studies have clearly identified the *A*. *gossypii* biotypes including cotton- and cucurbit-specialized aphids [4]. Host-specialization is ubiquitous among phytophagous insects [5–9]. Among the polyphagous aphids, not all individuals can feed on all of the putative host plants. Thus, populations are usually composed of subpopulations that can only feed on a few related plants. In particular, the specialization of sympatric populations to special host plants has been reported for many taxa in the Aphididae, such as the cotton—melon aphid *A*. *gossypii* Glover [10], the spiraea aphid *Aphis citicola* [11], the greenbug *Schizaphis graminum* (Rondani) [12] and the pea aphid *Acyrthosiphon pisum* [13]. Glinwood and Pettersson (2000) investigated the host choices and winter-host departure in *Rhopalosiphnum padi* (Linnaeus) under laboratory conditions, and indicated that the host alternation in *R*. *padi* was driven by individual and morph behavioral changes [14]. Studies on the correlated gene effects and habitat choices of pea aphids showed that the genetic architecture of traits associated with host selection may be a vital factor for the evolution of specialization [15]. Wu et al. (2013) used artificial host transfer experiments to study the host plant expansion of the host-specialized *A*. *gossypii* Glover, and concluded that zucchini and cowpea can induce the eating pattern changes in cotton- and cucurbit-specialized aphids [16]. Although previous studies have detected the dietary habit conversion of host-specialized *A*. *gossypii* under laboratory conditions, an actual field example of cotton-specialized aphids is still rare.

*A*. *gossypii* Glover exhibits strong fidelity to its host plants [17–19]. Host-specialization can help aphids to escape predators and avoid plant defenses [20–22]. However, cucurbits-specialized aphids may be exposed to a possible food deficiency when cucumber and zucchini crops become withered in the late autumn. Studies under laboratory conditions have shown the cucurbits-specialized aphids have the potential to feed on mature cotton plants and overcome food deficiency when the preferred host plant is absent [23]. However, in the field, whether cucurbit-specialized aphids can use the mature cotton to overcome a food deficiency remains ambiguous.

To fully understand the host plant expansion of host-specialized *A*. *gossypii* Glover, we used the following strategies: (i) we investigated the capacity of cotton-specialized clones to expand on a new plant species and used host transfer experiments to verify this ability in a field cage; (ii) further, we identified the biotypes of *A*.*gossypii* and investigated the relative frequencies of different biotypes occurring in a cotton field (mixed cotton and cucurbits), cotton farmland (cotton alone) and a field cage. Our study provides a field-based example of host plant expansion by the host-specialized *A*. *gossypii* Glover.

## Materials and methods

### Ethics statement

The cotton—melon aphid is a major insect pest with many host plants. No specific permits were required for the described field and laboratory studies. We confirm that the experiments in this study did not involve any endangered or protected species.

### Aphids and host plants

Cotton—melon aphids were collected from cotton fields at the Institute of Cotton Research (ICR) of the Chinese Academy of Agricultural Sciences (CAAS) in July 2013, and reared on cotton seedlings by parthenogenesis for 2 years in growth chambers (26 ± 1°C, L:D = 14:10, RH = 70%–80%). Three summer-host plants: cotton, cucumber and zucchini, were studied. The zucchini cultivars used to culture aphids were the conventional local breeds, and the cotton (except cotton in the cotton farmland) and cucumber cultivars were ‘CCRI 49’ and ‘Xinjin’, respectively. Seedlings of each plant were used for rearing aphids and for host transfer experiments in the laboratory.

### Experiment 1: Bioassay of cotton-specialized aphids

This experiment was conducted in Petri dishes in a growth chamber (26 ± 1°C, L: D = 14:10, 70%–80% RH) to measure the numbers of test aphids daily. A total of 10 to 15 apterous adult aphids were transplanted onto an excised leaf with the petiole wrapped in wet cotton wool. The next morning, only 10 first-stadium nymphs were kept on the leaf to initiate a cohort for establishing a life table. Thereafter, the survival and reproduction rates of the cohort were observed daily. During the reproductive period of the resulting adults, newborn aphids were recorded and removed daily, and this was continued until all of the adult aphids had died. The excised leaf was replaced by a fresh one at approximately every 2 days. Three to five cohorts of aphids were replicated for each life table. We used this method for aphids on cotton that we simultaneously cultured indoors. The same method was also used for aphids on cotton, cucumber and zucchini that were collected randomly from the field cage during August 2015 (for more information, see Experiment 2).

### Experiment 2: The population dynamics of cotton aphids in a field cage

One 18.0 m × 3.0 m × 2.7 m (length × width × height) field cage covered with 100-gauge mesh screen gauze was used in this experiment. The field cage was located at the ICR, CAAS farm (36.13°N, 114.85°E). Three types of host plants were randomly sown in rows (cotton: 7 rows, cucumber: 7 rows, zucchini: 6 rows) on April 25, in 2015. We selected three target plants for each host plant type. One week later, we sprayed imidacloprid (provided by Taian Health Chemical Co., Ltd., China) on all the host plants, according to the manufacturer’s instructions (70% imidacloprid WG, 45.0g/ ha.), to eliminate existing pests. Then, we transferred aphids from cotton in the laboratory to cotton and zucchini in the field cage on May 16; we transferred five adult aphids to each plant (3 host plant types 3 target plants). Aphids were surveyed every 3 days, and we counted the aphids on all leaves for cotton, but only on three leaves (top, middle and bottom) for cucumber and zucchini (both cucumber and zucchini were annual vine plants and hard to count for all the leaves). Aphids were surveyed from May 19 to September 9, 2015. During the survey, all plants were managed without spraying any additional chemical pesticides.

### Experiment 3: Identification of host-specialized aphids

We used the method of Wang [4] to identify the aphid biotypes. There were five single-nucleotide polymorphisms in the mtDNA gene fragment; these were T, A, A, T and T in cotton-specialized aphids and C, G, G, C, C in the cucurbit-specialized biotype. Total genomic DNA was extracted from single adult insects using the DNeasy Blood and Tissue Kit (Qiagen, Hilden, Germany) according to the manufacturer’s instructions. The PCR primers CytbF4 (5′-TACCATGAGGACAAATATCATTTTGA-3′) and 16SR2 (5′-AAGGGACGATAA GACCCTATAAAAC-3′) were used to amplify the sequence. Amplifications were conducted in 30-μL reaction volumes containing 3 μl of 10× ExTaq Buffer, 1.5 mM each dNTP, 6 μM each of primer, 0.75 U ExTaq DNA polymerase and 1 μL of template DNA under the following conditions: initial denaturation for 2 min at 94°C, followed by 35 cycles of 30 s at 94°C, 3 min at 64.5°C, and a final 10-min extension step at 72°C. The PCR products were examined under UV light after electrophoresis in 1.2% agarose. The qualified samples were sequenced in a single direction using the ABI 3730xl DNA Analyzer at GENEWIZ (Beijing, China).

### Experiment 4: Determining the proportions of two host-specialized aphids

Aphids were collected from the field cage (310 individuals), cotton field (621 individuals) and cotton farmland (581 individuals). The proportions of cotton-specialized and cucurbit-specialized aphids for the three locations were calculated based on Experiment 3. The field cage was introduced in Experiment 2. We collected aphids twice a month from June to August in 2015 for this experiment (S8 Table). Both the cotton field and cotton farmland were located at the ICR, CAAS farm (36.13°N, 114.85°E), and the sites were approximately 0.8 km apart. Some cucurbit crops planted by local farmers were located close to the cotton farmland. The 18 m × 6 m cotton field was divided randomly into nine plots and cotton, cucumber and zucchini were each planted in three of the plots in April 2015. Aphids were collected on May 13, May 15, May 27, June 21, August 5, August 21, September 9, September 22 and October 16, 2015 (S9 Table). No pesticides were sprayed during the study period. The cotton farmland was planted with a total of 4 hectares of cotton (with multiple varieties, CCRI 49, CCRI 45, CCRI 21, Simian 3, Shiyuan 321, etc., planted randomly within the plots, and 112 rows in total). We treated them all the same and collected samples from the thirty randomly selected rows at the peak of cotton aphid occurrence (July 22 and September 8 in 2015 and May 20, 2016) and during the period of cucurbits senescence (September 8 and October 14 in 2015; S10 Table). The same sample plants were visited at each sampling date. For all sampling sites, only one individual per plant was collected to avoid sampling the offspring of a single female. All samples were collected randomly and stored at –80°C until DNA extraction.

## Statistical analyses

A generalized linear model (GzLM) with binomial distribution and logit link function was conducted to compare survival and percentage data. A post-hoc-test of pairwise comparisons was continued to compare the difference of different host transfer type if the linear model yields a significant result. Life-table parameters of cotton aphids were compared by post-hoc Tukey’s Honestly Significant Difference (HSD) method based on One way ANOVA. The life-table parameters of aphids on cotton, cucumber and zucchini, including *R*_*0*_, average generation time (*T*) and *r*_*m*_, were calculated using the following formulas: *R*_*0*_ = ∑ *lxmx*, *T* = (∑ *xlxmx*)/(∑ *lxmx*) and *r*_*m*_ = (*ln R*_*0*_)/*T*. In these equations, the age-specific survival rate (*lx*) is the proportion of individuals in the original cohort alive at age *x* (days) and the age-specific fecundity (*mx*) is the mean number of female progeny produced per female alive on day *x*. The biotypes of aphids were aligned with DNAMAN 8. For Experiment 2, numbers of aphids were converted into mean number per 100 plants. Data were Lg10-transformed if needed to meet the assumptions of normality and homoscedasticity required for the One way ANOVA analyses. All those statistical analyses were performed with SPSS (version 20 for Windows 2007).

## Results

### Survival and life table parameters of cotton-specialized aphids

**Fig 1:**
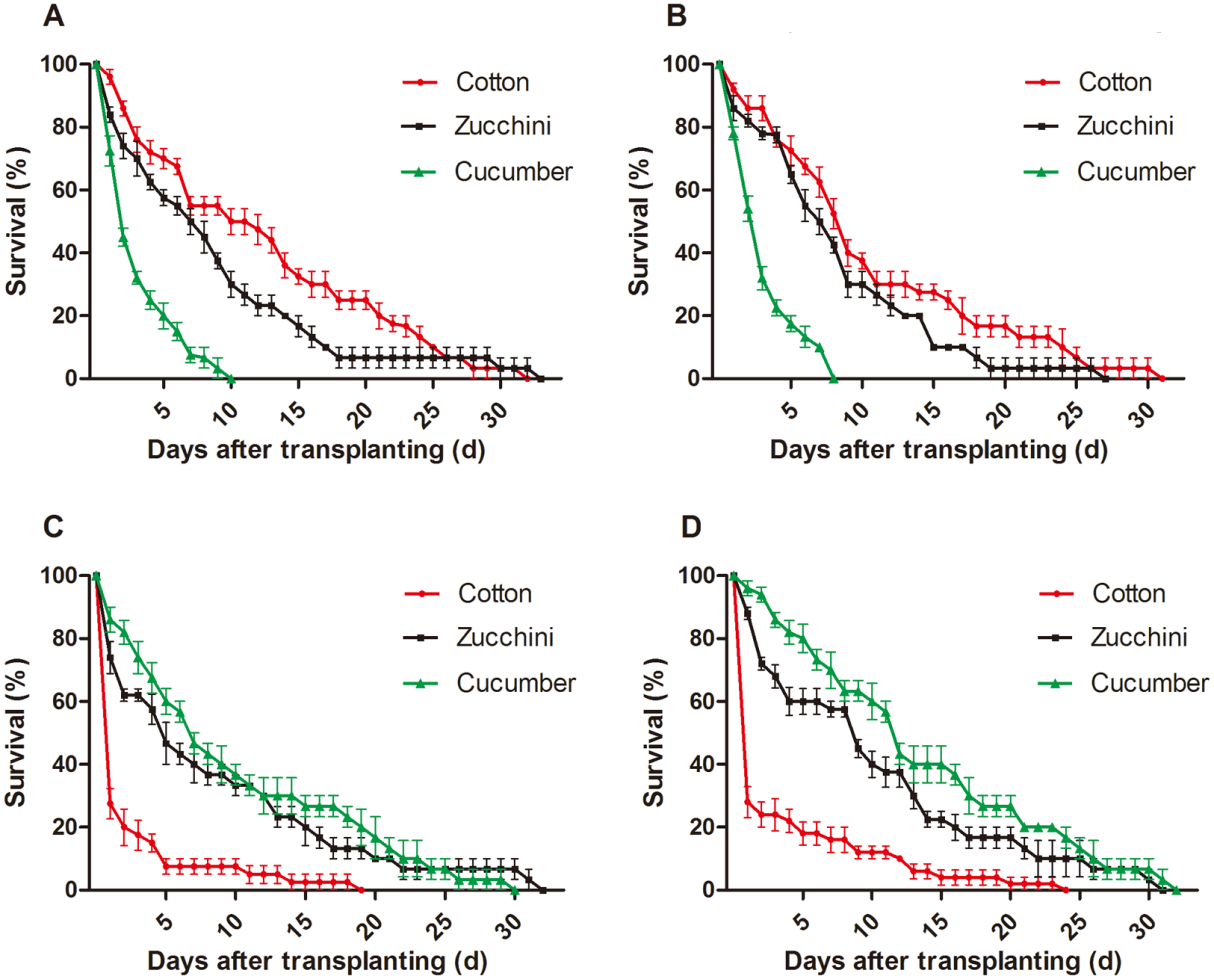
Survival (± SEM) curves of aphids after transferring to cotton, cucumber and zucchini. (A) Aphids from cotton in the laboratory. (B) Aphids from cotton in the field cage. (C) Aphids from cucumber in the field cage. (D) Aphids from zucchini in the field cage.

After transferring from cotton to cucumber, aphids’ offspring survival rates decreased rapidly, with only17.5 ± 2.5% and 20.0 ± 4.1% of offspring surviving on the 5th day in the field cage and the laboratory, respectively (Fig 1A and 1B; S1 Table). Aphids that shifted from cucumber onto cotton also displayed similar results, with only 7.5 ± 2.5% of aphids surviving on the 5th day in the field cage (Fig 1C). Approximately 72.5 ± 4.8% and 65.0 ± 2.9% of aphids from cotton in the field cage were alive on cotton and zucchini, respectively, by the 5th day (S2 Table). Aphids from cucumber in the field also showed the same tendency, with higher survival rates when they transferred to cucumber (S3 Table). When aphids from zucchini transferred, 18.0 ± 3.7%, 80.0 ± 4.5% and 60.0 ± 4.1% survived on cotton, cucumber and zucchini, respectively, on the 5th day (Fig 1D; S4 Table). Aphids from cotton or cucumber had a preference for their natal host, while aphids on zucchini exhibited a stronger preference for cucumber.

The life-table parameters of aphids in different treatments also indicated similar results. In the laboratory, the net reproductive rate, *R*_*0*_ (25.88 ± 1.26), and the intrinsic rate of increase, *r*_*m*_ (0.26 ± 0.02), after transfer of aphids from cotton to cotton, were significantly (post-hoc Tukey’s HSD test: *R*_*0*_, P = 0.00; *r*_*m*_, P = 0.00) higher than those of the same aphids transferred to cucumber (0.73 ± 0.13 and −0.10 ± 0.05, respectively) (S5 Table). In the field cage, aphids transferred from cotton to the two other host plants displayed the same tendency, with *R*_*0*_ (23.67 ± 1.68), *T* (12.62 ± 0.42) and *r*_*m*_ (0.25 ± 0.01) on cotton, and *R*_*0*_ (0.87 ± 0.06), *T* (3.93 ± 0.70) and *r*_*m*_ (−0.04 ± 0.02) on cucumber (S6 Table). Aphids from cucumber showed the opposite result when transferred to cotton or cucumber; their *R*_*0*_ (0.92± 0.10), *T* (9.04 ± 0.19) and *r*_*m*_ (−0.01 ± 0.01) on cotton were significantly (*R*_*0*_, *T* and *r*_*m*_: P < 0.05) different from their *R*_*0*_ (19.91± 1.85), *T* (12.96 ± 0.11) and *r*_*m*_ (0.23 ± 0.01) on cucumber (S7 Table). Compared with the above groups, aphids from zucchini show a significant preference for cucumber, while the other two groups showed a preference for their natal host plants. The *R*_*0*_ (31.94 ± 0.02), *T* (12.89 ± 0.04) and *r*_*m*_ (0.27 ± 0.00) for aphids transferred to cucumber were higher than those on cotton (significantly) or zucchini (not significant) (Table 1).

**Table 1:** Life-table parameters of aphids transferred from zucchini (in the field cage) to three host plants.

Host transfer type	Net reproductive rate *R*_*0*_	Average generation time *T*	Intrinsic rate of increase *r*_*m*_
Zucchini—cotton	4.86 ± 1.09b	8.96 ± 0.68b	0.17 ± 0.02b
Zucchini—zucchini	21.06 ± 0.05a	12.63 ± 0.06a	0.24 ± 0.00a
Zucchini—cucumber	31.94 ± 0.02a	12.89 ± 0.04a	0.27 ± 0.00a
Statistics	*F* = 60.16/ *p* = 0.00	*F* = 30.99/ *p* = 0.00	*F* = 14.59/ *p* = 0.01

Note: Data are Means ± SE. Statistical significance based on One way ANOVA test. Values in the same column followed by different letters are significantly different at P < 0.05 according to the post-hoc Tukey’s HSD method.

### Population dynamics of cotton aphids in the field cage

**Fig 2:**
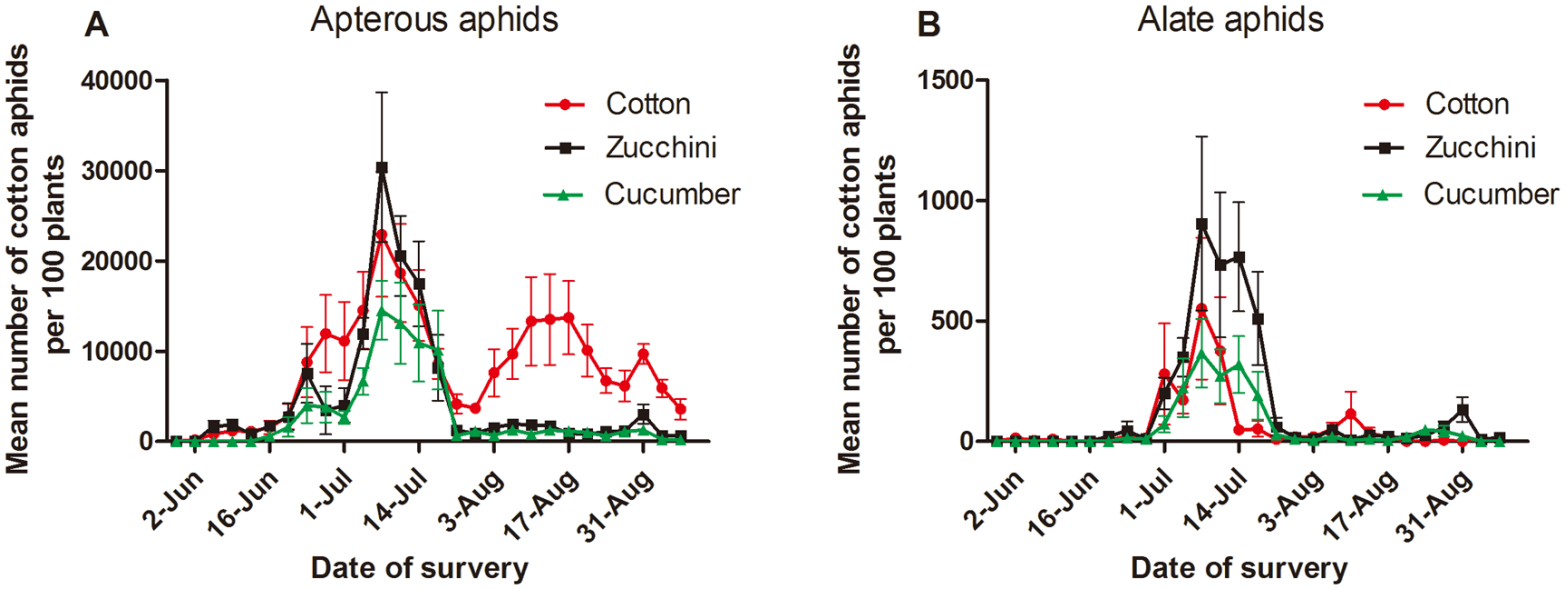
Mean population dynamics (± SEM) of cotton aphids per 100 plants surveyed in the field cage. (A) Population dynamics of apterous aphids in the field cage. (B) Population dynamics of alate aphids in the field cage.

As shown in Fig 2A, the apterous aphid population on cotton peaked on July 7 and August 17 in 2015, and their numbers were 22,933 and 13,704 (per 100 plants). The highest mean number of apterous aphids (30361 per 100 plants) was recorded on zucchini at July 7 in 2015. Apterous aphids were first discovered on cucumber on June 12 and and their number increased exponentially thereafter. The total number of individuals reached a peak of 14,533 (per 100 plants) on July 7 and then gradually decreased. For alate aphids on cotton and zucchini, the numbers of individuals peaked on July 7, at 552 and 905 (per 100 plants), respectively. For alate aphids on cucumber, individuals were first recorded on June 23 and their number increased gradually, reaching a peak of 366 (per 100 plants) on July 7 (Fig 2B).

### Host-specialized aphid identification

A total of 1512 aphids were collected and sequenced. The result of comparing sequences revealed that 1494 individuals were *A*. *gossypii*. Only one, nine and eight individuals of other species were collected in the field cage, cotton field and cotton farmland respectively. *A*. *gossypii* included two biotypes, cotton-specialized biotype and cucurbit-specialized, and the relative percentages varied among the sample sites and other different sampling dates (S8, S9 and S10 Tables).

### Proportions of the two host-specialized aphids

**Fig 3:**
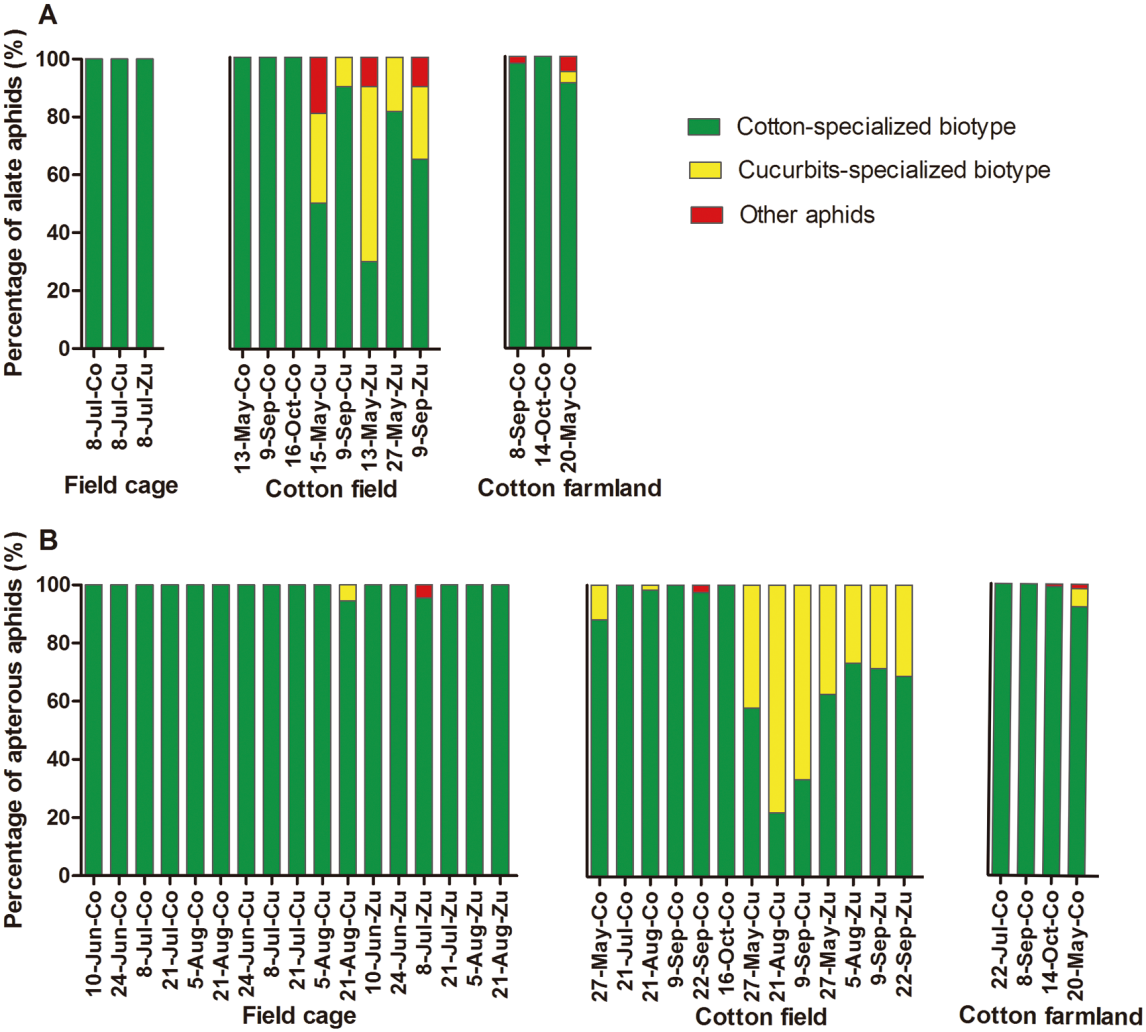
Percentage of two host biotypes of *A*. *gossypii* on different host plants in three different places. The horizontal axis shows the date of sampling and the host plants in three different locations: Co, cotton; Cu, cucumber, and Zu, zucchini. All samples were collected in 2015, except for individuals collected from cotton farmland on May 20, 2016.

In the field cage, the vast majority (99.4%) of aphids were cotton-specialized. The cucurbit-specialized biotype was only found on cucumber on August 21, and comprised 5.6% of the total individuals (Fig 3).

On cotton plants in the cotton field, all of the alate aphids and a majority (98.0%) of apterous individuals were cotton-specialized (Fig 3). The cucurbit-specialized biotype comprised a minority (11.8%) of the population on May 27, 2015. Among the alate aphids on cucumber, 50.0% and 30.8% were cotton-specialized and cucurbit-specialized biotype, respectively, were on May 15. For the same group on September 9, 2015, 90.0% and 10.0% were cucurbit- and cotton-specialized individuals, respectively. For apterous aphids on cucumber, 42.1%, 78.1% and 66.7% were cucurbit-specialized on May 27, August 21 and September 9, respectively. On zucchini, the proportion of the cotton-specialized biotype (30.0% of the alate aphids) was lower than that of the cucurbit-specialized biotype (60.0% of the alate aphids) on May 13. However, on the other dates in 2015, cotton-specialized aphids accounted for larger proportions, with 81.5% and 65.0% on May 27 and September 9 for alate aphids, and 62.5%, 73.3%, 71.4% and 68.8% on May 27, August 5, September 9 and September 22 for apterous aphids (Fig 3). Overall, the difference in the proportion of the two biotypes proportion between cucumber and zucchini was as follows: on cucumber, there were significantly (GzLM: *χ*^*2*^ = 35.432, *df* = 1, *p* = 0.000) more cucurbit-specialized apterous aphids (62.3%), and on zucchini there were significantly (GzLM: *χ*^*2*^ = 35.432, *df* = 1, *p* = 0.000) more cotton-specialized apterous aphids (69.0%); there were no significant differences for alate aphids (Table 2).

**Table 2:** Percentage of host-specialized biotype from cucumber and zucchini in cotton field.

Biotype/ host	Cotton-specialized biotype (%)		Cucurbits-specialized biotype (%)	
	Alate	Apterous	Alate	Apterous
Cucumber	70.0 ± 20.0	37.7 ± 10.6	20.0 ± 10.4	62.3 ± 10.6
Zucchini	58.8 ± 15.2	69.0 ± 2.4	34.5 ± 12.9	31.0 ± 2.4
Statistics	*χ*^*2*^ = 0.365/ *df* = 1/ *p* = 0.546	***χ***^***2***^ = **35.432/** ***df* = 1/** ***p* = 0.000**	*χ*^*2*^ = 0.106/ *df* = 1/ *p* = 0.744	***χ***^***2***^ = **35.432/** ***df* = 1/** ***p* = 0.000**

Note: Data are Means ± SE, *χ*^*2*^ = Likelihood ratio Chi-Square. And data were analyzed by GzLM with binomial distribution and logit link function (the significant statistics were marked in bold).

In cotton farmland, 97.4% of individuals were cotton-specialized, and only 1.2% belonged to the cucurbit-specialized biotype (Fig 3). In 2015, all of the aphids on cotton aphids were cotton-specialized for all sampling dates (in July, September and October). On May 20, 2016, 91.0% of alate aphids and 92.3% of apterous individuals were recorded were found to be cotton-specialized. On the same date, cucurbit-specialized aphids accounted for just 3.8% and 6.2% of alate and apterous individuals, respectively.

## Discussion

The evolutionary direction of host-specialization varies in phytophagous insects. Some species evolve toward monophagy or oligophagy, while others evolve toward polyphagy [24–26]. Polyphagy is most likely ancestral to monophagy and oligophagy in terms of host shift [27, 28]. There are several hypotheses for the evolution of host alternation, such as optimization of nutritional conditions by seasonally complementary host-plant growth [29, 30], spawning grounds [31], temperature limits [32], escape from predators [33, 34], avoiding related host-plant defenses [35, 36], and restriction of ancestral winter hosts [37]. Moran (1988) compared those hypotheses and argued that specialization on a beneficial, narrow ecological niche is an evolutionary dead end that restricts future evolution and ultimately increases the probability of extinction [38]. However, the butterfly tribe Nymphalini exhibited a dynamic model for changes in their host range, and there seemed to be no directionality in the host range evolution toward further specialization [16, 39]. Additionally, our studies suggest that zucchini can induce the conversion of eating patterns in cotton-specialized aphids, which ultimately broke the host-specialization. We provide an actual example to support the ‘oscillation hypothesis’, and supply evidence for the view that specialization does not always represent an evolutionary dead end.

The classic ‘oscillation hypothesis’ provides a comprehensive explanation for how lineages of phytophagous insect could alternate between generalist and specialist phases [40]. Specialization may evolve toward the specialist, and then undergo the stages of host and geographical expansion, and ultimately evolve to fit local conditions. While local host plant adaptation and host expansion could lead to the formation of specialization and speciation, the local host plant specialization should often reoccur because of the female’s maximum fitness. Together, phytophagous insects and their host plants compose one of the most ubiquitous groups, and their specialization can increase the likelihood of population subdivision and speciation, not the dead end of evolution.

Our recorded data showed the cotton-specialized clones can expand on a new plant species under environmental stress (in the field cage). After transferring to cotton and zucchini, about 2 weeks later, aphids were also recorded on cucumber and increased exponentially in the field cage. The results of biotype in the field cage showed majority of individuals were belonged to cotton-specialized biotype, and the minority of cucurbit-specialized aphids and other aphids may brought from human clothes during the process of investigation. Previous studies have reported the cotton-specialized aphids could not use cucumber directly, but could use zucchini as the intermediate host plant [4, 16]. Thus, aphids on cucumber in our study could only come from zucchini. The bioassay experiment provided strong evidence for this point. Our survival data and life-table parameters demonstrated the offspring of aphids from cotton in the field cage can establish populations on cotton leaves but cannot survive on cucumber leaves, and individuals from cucumber can establish populations on cucumber leaves but cannot survive on cotton leaves.

However, *A*. *gossypii* from cotton and cucumber maintained their specialized preferences in the field (under low environmental pressure). In the cotton field, the proportion of apterous cotton-specialized aphids on cotton was greater, and the percentage of cucurbit-specialized aphids on cucumber was greater. All of the alate individuals on cotton were the cotton-specialized biotype. On May 15 and September 19, 50.0% and 90.0%, respectively, of the alate aphids on cucumber were cotton-specialized clones. This may have been a result of the population peak in cotton aphids occurring on cotton in the field (high environmental pressure). In the cotton farmland, 97.4% of aphids belonged to the cotton-specialized biotype and only 1.2% were the cucurbit-specialized biotype.

The fitness of *A*. *gossypii* changed after transfer to cotton and zucchini, with the cotton-specialized clones on zucchini having a stronger preference for cucumber. The bioassay experiment showed that aphids from zucchini that transferred to cucumber had a higher survival rate (80.0 ± 4.5%) and higher life table parameters (*R*_*0*_ = 31.94 ± 0.02, *T* = 12.89 ± 0.04 and *r*_*m*_ = 0.27 ± 0.00).

Zucchini was the intermediate host plant between cotton and cucumber, which may signal a vital role in the process of specialization between cotton- and cucurbit-specialized aphids. Both of the biotypes coexisted on zucchini in the cotton field, and the cotton-specialized biotype made up a larger proportion (69.0%) of apterous aphids. A previous laboratory-based study reported that the presence of zucchini can alter the preference of *A*. *gossypii* for cotton and cucumber [16]. Zheng (2007) investigated the fitness and reciprocal transfer pathways of the two host biotypes under laboratory condition, and concluded that cotton- and cucurbit-specialized aphids were able to transfer between cucumber and cotton plants via the intermediate host zucchini [41]. In our current study, zucchini induced host alternation of the cotton- and cucurbit-specialized biotypes, which displayed reversibility in specialization. The switch we found in *A*. *gossypii* individuals from specialized to generalized via zucchini as an intermediate host plant provides direct evidence for the ‘oscillation hypothesis’. However, the actual mechanism behind this switch requires further study, as does the question of how cucurbit-specialized aphids are able to use cotton via an intermediate plant in the fields. Additionally, individuals collected from cotton farmland included a minority of cucurbits-specialized aphids on May 20 in 2016, and none were recorded on July 22, September 8 and October 14 in 2015. This may have been a result of our relatively small sample sizes compared with previous studies. The local cucurbit crops generally withered in early September, and some zucchini and cucumber plants owned by local farmers were near to our experimental site. This may have affected the results to a small degree. The relative proportions of the different biotypes on cotton farmland seem to suggest that most cucurbit-specialized clones were not able to feed on cotton as an alternative to cucurbits. Because zucchini is often present together with cotton and cucumber in fields and greenhouses, eliminating intermediate host plants is likely to be effective in suppressing *A*. *gossypii* outbreaks.

## Supporting information

S1 Table Survival of aphids after transferring from cotton (in the laboratory) to summer hosts. (DOCX)

S2 Table Survival of aphids after transferring from cotton (in the field cage) to summer hosts. (DOCX)

S3 Table Survival of aphids after transferring from cucumber (in the field cage) to summer hosts. (DOCX)

S4 Table Survival of aphids after transferring from zucchini (in the field cage) to summer hosts. (DOCX)

S5 Table Life-table parameters of aphids transferred from cotton (in the laboratory) to summer hosts. (DOCX)

S6 Table Life-table parameters of aphids transferred from cotton (in the field cage) to summer hosts. (DOCX)

S7 Table Life-table parameters of aphids transferred from cucumber (in the field cage) to summer hosts. (DOCX)

S8 Table Numbers of two *A*. *gossypii* host biotypes on different host plants in the field cage. (DOCX)

S9 Table Numbers of two *A*. *gossypii* host biotypes on different host plants in the cotton field. (DOCX)

S10 Table Numbers of two *A*. *gossypii* host biotypes on cotton in the cotton farmland. (DOCX)
