# Supplementary material for: An example of host plant expansion of host-specialized Aphis gossypii Glover in the field
Source: PLoS One. 2017 May 17;12(5):e0177981. doi: 10.1371/journal.pone.0177981 (PMC5435340; doi:10.1371/journal.pone.0177981)
Supplement: S1 Table — (DOCX) [file pone.0177981.s001.docx]

**S1 Table. Survival of aphids after transferring from cotton (in the laboratory) to summer hosts.**

| Host transfer type | Survival (%) | | | | | |
| --- | --- | --- | --- | --- | --- | --- |
|  | 1d | 2d | 3d | 4d | 5d | 6d |
| Cotton-cotton | 96.0 ±  2.4a | 86.0 ±  2.4a | 76.0 ±  4.0a | 72.0 ±  3.7a | 70.0 ±  3.2a | 67.5 ±  2.5a |
| Cotton-zucchini | 84.0 ±  2.4ab | 74.0 ±  4.0a | 70.0 ±  5.5a | 62.5 ±  2.5a | 62.5 ±  2.5 a | 55.0 ±  2.9b |
| Cotton-cucumber | 72.5 ±  4.8b | 45.0 ±  2.9c | 32.0 ±  2.0b | 25.0 ±  2.9b | 20.0 ±  4.1b | 15.0 ±  2.9c |
| Statistics | *χ^2^* = 10.544/  *df* = 2/  *p* = 0.005 | *χ^2^* = 18.189/  *df* = 2/  *p* = 0.000 | *χ^2^* = 20.061/  *df* = 2/  *p* = 0.000 | *χ^2^* =  21.902/  *df* = 2/  *p* = 0.000 | *χ^2^* = 24.521/  *df* = 2/  *p* = 0.000 | *χ^2^* = 26.207/  *df* = 2/  *p* = 0.000 |

Note: Data are Means ± SE, *χ^2^* = Likelihood ratio Chi-Square. Statistical significance based on GzLM with binomial distribution and logit link function. Values in the same column followed by different letters are significantly different at P < 0.05 according to the post-hoc-test of pairwise comparisons.
