# Supplementary material for: An example of host plant expansion of host-specialized Aphis gossypii Glover in the field
Source: PLoS One. 2017 May 17;12(5):e0177981. doi: 10.1371/journal.pone.0177981 (PMC5435340; doi:10.1371/journal.pone.0177981)
Supplement: S2 Table — (DOCX) [file pone.0177981.s002.docx]

**S2 Table. Survival of aphids after transferring from cotton (in the field cage) to summer hosts.**

| Host transfer type | Survival (%) | | | | | |
| --- | --- | --- | --- | --- | --- | --- |
|  | 1d | 2d | 3d | 4d | 5d | 6d |
| Cotton-cotton | 92.0 ±  2.0 | 86.0 ±  4.0a | 86.0 ±  4.0a | 76.0 ±  2.4a | 72.5 ±  4.8a | 67.5 ±  2.5a |
| Cotton-zucchini | 86.0 ±  4.0 | 82.0 ±  2.0a | 78.0 ±  2.0a | 77.5 ±  2.5a | 65.0 ±  2.9 a | 55.0 ±  5.0a |
| Cotton-cucumber | 78.0 ±  2.0 | 54.0 ±  4.0b | 32.0 ±  3.7b | 22.5 ±  2.5b | 17.5 ±  2.5b | 13.3 ±  3.3b |
| Statistics | *χ^2^* =  4.001/  *df* = 2/  *p* = 0.135 | *χ^2^* = 15.287/  *df* = 2/  *p* = 0.000 | *χ^2^* = 37.734/  *df* = 2/  *p* = 0.000 | *χ^2^* =  34.569/  *df* = 2/  *p* = 0.000 | *χ^2^* =  30.275/  *df* = 2/  *p* = 0.000 | *χ^2^* = 23.289/  *df* = 2/  *p* = 0.000 |

Note: Data are Means ± SE, χ2 = Likelihood ratio Chi-Square. Statistical significance based on GzLM with binomial distribution and logit link function. Values in the same column followed by different letters are significantly different at P < 0.05 according to the post-hoc-test of pairwise comparisons.
