# Supplementary material for: An example of host plant expansion of host-specialized Aphis gossypii Glover in the field
Source: PLoS One. 2017 May 17;12(5):e0177981. doi: 10.1371/journal.pone.0177981 (PMC5435340; doi:10.1371/journal.pone.0177981)
Supplement: S3 Table — (DOCX) [file pone.0177981.s003.docx]

**S3 Table. Survival of aphids after transferring from cucumber (in the field cage) to summer hosts.**

| Host transfer type | Survival (%) | | | | | |
| --- | --- | --- | --- | --- | --- | --- |
|  | 1d | 2d | 3d | 4d | 5d | 6d |
| Cucumber-cotton | 27.5 ±  4.8b | 20.0 ±  5.8b | 17.5 ±  4.8b | 15.0 ±  2.9b | 7.5 ±  2.5b | 7.5 ±  2.5c |
| Cucumber-zucchini | 74.0 ±  5.1a | 62.0 ±  2.0a | 62.0 ±  2.0a | 57.5 ±  4.8a | 46.7 ± 6.7a | 43.3 ± 3.3b |
| Cucumber-cucumber | 86.0 ±  4.0a | 82.0 ±  3.7a | 74.0 ±  5.1a | 67.5 ±  4.8a | 60.0 ± 4.1a | 56.7 ± 3.3a |
| Statistics | *χ^2^* =  36.430/  *df* = 2/  *p* = 0.000 | *χ^2^* = 37.636/  *df* = 2/  *p* = 0.000 | *χ^2^* = 34.439/  *df* = 2/  *p* = 0.000 | *χ^2^* = 27.010/  *df* = 2/  *p* = 0.000 | *χ^2^* = 28.000/  *df* = 2/  *p* = 0.000 | *χ^2^* = 23.417/  *df* = 2/  *p* = 0.000 |

Note: Data are Means ± SE, *χ^2^* = Likelihood ratio Chi-Square. Statistical significance based on GzLM with binomial distribution and logit link function. Values in the same column followed by different letters are significantly different at P < 0.05 according to the post-hoc-test of pairwise comparisons.
