# Supplementary material for: An example of host plant expansion of host-specialized Aphis gossypii Glover in the field
Source: PLoS One. 2017 May 17;12(5):e0177981. doi: 10.1371/journal.pone.0177981 (PMC5435340; doi:10.1371/journal.pone.0177981)
Supplement: S4 Table — (DOCX) [file pone.0177981.s004.docx]

**S4 Table. Survival of aphids after transferring from zucchini (in the field cage) to summer hosts.**

| Host transfer type | Survival (%) | | | | | |
| --- | --- | --- | --- | --- | --- | --- |
|  | 1d | 2d | 3d | 4d | 5d | 6d |
| Zucchini-cotton | 28.0 ±  4.9b | 24.0 ±  4.0c | 24.0 ±  5.1c | 22.0 ±  3.7c | 18.0 ±  3.7c | 18.0 ±  3.7b |
| Zucchini-zucchini | 88.0 ±  2.0a | 72.0 ±  2.0b | 68.0 ±  3.7b | 60.0 ±  4.5b | 60.0 ±  4.1b | 60.0 ±  4.1a |
| Zucchini-cucumber | 96.0 ±  2.4a | 94.0 ±  2.4a | 86.0 ±  2.4a | 82.0 ±  3.7a | 80.0 ±  4.5a | 73.3 ±  3.3a |
| Statistics | *χ^2^* =  64.575/  *df* = 2/  *p* = 0.000 | *χ^2^* =  60.047/  *df* = 2/  *p* = 0.000 | *χ^2^* = 44.395/  *df* = 2/  *p* = 0.000 | *χ^2^* = 39.504/  *df* = 2/  *p* = 0.000 | *χ^2^* = 42.803/  *df* = 2/  *p* = 0.000 | *χ^2^* = 29.746/  *df* = 2/  *p* = 0.000 |

Note: Data are Means ± SE, *χ^2^* = Likelihood ratio Chi-Square. Statistical significance based on GzLM with binomial distribution and logit link function. Values in the same column followed by different letters are significantly different at P < 0.05 according to the post-hoc-test of pairwise comparisons.
