# Supplementary material for: An example of host plant expansion of host-specialized Aphis gossypii Glover in the field
Source: PLoS One. 2017 May 17;12(5):e0177981. doi: 10.1371/journal.pone.0177981 (PMC5435340; doi:10.1371/journal.pone.0177981)
Supplement: S5 Table — (DOCX) [file pone.0177981.s005.docx]

**S5 Table. Life-table parameters of aphids transferred from cotton (in the laboratory) to summer hosts.**

| Host transfer type | Net reproductive rate *R_0_* | Average generation time *T* | Intrinsic rate of increase *r_m_* |
| --- | --- | --- | --- |
| Cotton–cotton | 25.88 ± 1.26a | 12.81 ± 0.69a | 0.26 ± 0.02a |
| Cotton–zucchini | 17.66 ± 1.57b | 11.76 ± 0.47a | 0.24 ± 0.16a |
| Cotton–cucumber | 0.73 ± 0.13c | 3.49 ± 0.11b | -0.10 ± 0.05b |
| Statistics | *F* = 120.88/  *p* = 0.00 | *F* = 110.82/  *p* = 0.00 | *F* = 35.12/  *p* = 0.00 |

Note: Data are Means ± SE. Statistical significance based on One way ANOVA test.

Values in the same column followed by different letters are significantly different at P < 0.05 according to the post-hoc Tukey’s HSD method.
