# Supplementary material for: An example of host plant expansion of host-specialized Aphis gossypii Glover in the field
Source: PLoS One. 2017 May 17;12(5):e0177981. doi: 10.1371/journal.pone.0177981 (PMC5435340; doi:10.1371/journal.pone.0177981)
Supplement: S6 Table — (DOCX) [file pone.0177981.s006.docx]

**S6 Table. Life-table parameters of aphids transferred from cotton (in the field cage) to summer hosts.**

| Host transfer type | Net reproductive rate *R_0_* | Average generation time *T* | Intrinsic rate of increase *r_m_* |
| --- | --- | --- | --- |
| Cotton–cotton | 23.67 ± 1.68a | 12.62 ± 0.42a | 0.25 ± 0.01a |
| Cotton–zucchini | 14.72 ± 0.33b | 10.15 ± 0.09b | 0.27 ± 0.00a |
| Cotton–cucumber | 0.87 ± 0.06c | 3.93 ± 0.70c | -0.04 ± 0.02b |
| Statistics | *F* = 1018.68/  *p* = 0.00 | *F* = 89.21/  *p* = 0.00 | *F* = 175.29/  *p* = 0.00 |

Note: Data are Means ± SE. Statistical significance based on One way ANOVA test.

Values in the same column followed by different letters are significantly different at P < 0.05 according to the post-hoc Tukey’s HSD method.
