# Supplementary material for: An example of host plant expansion of host-specialized Aphis gossypii Glover in the field
Source: PLoS One. 2017 May 17;12(5):e0177981. doi: 10.1371/journal.pone.0177981 (PMC5435340; doi:10.1371/journal.pone.0177981)
Supplement: S7 Table — (DOCX) [file pone.0177981.s007.docx]

**S7 Table. Life-table parameters of aphids transferred from cucumber (in the field cage) to summer hosts.**

| Host transfer type | Net reproductive rate *R_0_* | Average generation time *T* | Intrinsic rate of increase *r_m_* |
| --- | --- | --- | --- |
| Cucumber–cotton | 0.92 ± 0.10c | 9.04 ± 0.19c | -0.01 ± 0.01b |
| Cucumber–zucchini | 13.36 ± 0.67b | 11.57 ± 0.02b | 0.22 ± 0.00a |
| Cucumber–cucumber | 19.91 ± 1.85a | 12.96 ± 0.11a | 0.23 ± 0.01a |
| Statistics | *F* = 382.39/  *p* = 0.00 | *F* = 252.32/  *p* = 0.00 | *F* = 307.96/  *p* = 0.00 |

Note: Data are Means ± SE. Statistical significance based on One way ANOVA test.

Values in the same column followed by different letters are significantly different at P < 0.05 according to the post-hoc Tukey’s HSD method.
