# Supplementary material for: An example of host plant expansion of host-specialized Aphis gossypii Glover in the field
Source: PLoS One. 2017 May 17;12(5):e0177981. doi: 10.1371/journal.pone.0177981 (PMC5435340; doi:10.1371/journal.pone.0177981)
Supplement: S8 Table — (DOCX) [file pone.0177981.s008.docx]

**S8 Table. Numbers of two *A. gossypii* host biotypes on different host plants in the field cage.**

| Date of sampling | Host plants | Alate/apterous aphids | Total number | Numbers of the cotton-specialized biotype | Numbers of the cucurbits-specialized biotype | Numbers of other aphids |
| --- | --- | --- | --- | --- | --- | --- |
| Jun.10 | Cotton | Apterous | 7 | 7 | 0 | 0 |
|  | Zucchini | Apterous | 8 | 8 | 0 | 0 |
| Jun.24 | Cotton | Apterous | 9 | 9 | 0 | 0 |
|  | Cucumber | Apterous | 8 | 8 | 0 | 0 |
|  | Zucchini | Apterous | 9 | 9 | 0 | 0 |
| Jul.8 | Cotton | Alate | 21 | 21 | 0 | 0 |
|  | Zucchini | Alate | 23 | 23 | 0 | 0 |
|  | Cucumber | Alate | 22 | 22 | 0 | 0 |
|  | Cotton | Apterous | 19 | 19 | 0 | 0 |
|  | Cucumber | Apterous | 15 | 15 | 0 | 0 |
|  | Zucchini | Apterous | 22 | 21 | 0 | 1 |
| Jul.21 | Cotton | Apterous | 17 | 17 | 0 | 0 |
|  | Cucumber | Apterous | 18 | 18 | 0 | 0 |
|  | Zucchini | Apterous | 18 | 18 | 0 | 0 |
| Aug.5 | Cotton | Apterous | 10 | 10 | 0 | 0 |
|  | Cucumber | Apterous | 24 | 24 | 0 | 0 |
|  | Zucchini | Apterous | 9 | 9 | 0 | 0 |
| Aug.21 | Cotton | Apterous | 11 | 11 | 0 | 0 |
|  | Cucumber | Apterous | 18 | 17 | 1 | 0 |
|  | Zucchini | Apterous | 22 | 22 | 0 | 0 |
