# Supplementary material for: An example of host plant expansion of host-specialized Aphis gossypii Glover in the field
Source: PLoS One. 2017 May 17;12(5):e0177981. doi: 10.1371/journal.pone.0177981 (PMC5435340; doi:10.1371/journal.pone.0177981)
Supplement: S9 Table — (DOCX) [file pone.0177981.s009.docx]

**S9 Table. Numbers of two *A. gossypii* host biotypes on different host plants in the cotton field.**

| Date of sampling | Host plants | Alate/  apterous aphids | Total number | Numbers of the cotton-specialized biotype | Numbers of the cucurbits-specialized biotype | Numbers of other aphids |
| --- | --- | --- | --- | --- | --- | --- |
| May.13 | Cotton | Alate | 6 | 6 | 0 | 0 |
|  | Zucchini | Alate | 10 | 3 | 6 | 1 |
| May.15 | Cucumber | Alate | 26 | 13 | 8 | 5 |
| May.27 | Zucchini | Alate | 27 | 22 | 5 | 0 |
|  | Cotton | Apterous | 17 | 15 | 2 | 0 |
|  | Cucumber | Apterous | 19 | 11 | 8 | 0 |
|  | Zucchini | Apterous | 24 | 15 | 9 | 0 |
| Jul.21 | Cotton | Apterous | 13 | 13 | 0 | 0 |
| Aug.5 | Zucchini | Apterous | 60 | 44 | 16 | 0 |
| Aug.21 | Cotton | Apterous | 57 | 56 | 1 | 0 |
|  | Cucumber | Apterous | 64 | 14 | 50 | 0 |
| Sep.9 | Cotton | Alate | 71 | 71 | 0 | 0 |
|  | Cucumber | Alate | 10 | 9 | 1 | 0 |
|  | Zucchini | Alate | 20 | 13 | 5 | 2 |
|  | Cotton | Apterous | 49 | 49 | 0 | 0 |
|  | Cucumber | Apterous | 18 | 6 | 12 | 0 |
|  | Zucchini | Apterous | 21 | 15 | 6 | 0 |
| Sep.22 | Cotton | Apterous | 39 | 38 | 0 | 1 |
|  | Zucchini | Apterous | 16 | 11 | 5 | 0 |
| Oct.16 | Cotton | Alate | 26 | 26 | 0 | 0 |
|  | Cotton | Apterous | 28 | 28 | 0 | 0 |
