# Supplementary material for: An example of host plant expansion of host-specialized Aphis gossypii Glover in the field
Source: PLoS One. 2017 May 17;12(5):e0177981. doi: 10.1371/journal.pone.0177981 (PMC5435340; doi:10.1371/journal.pone.0177981)
Supplement: S10 Table — (DOCX) [file pone.0177981.s010.docx]

**S10 Table. Numbers of two *A. gossypii* host biotypes on cotton in the cotton farmland.**

| Date of sampling | Host plants | Alate/apterous aphids | Total number | Numbers of the cotton-specialized biotype | Numbers of the cucurbits-specialized biotype | Numbers of other aphids |
| --- | --- | --- | --- | --- | --- | --- |
| Jul.22  (2015) | Cotton | Apterous | 66 | 66 | 0 | 0 |
| Sep.8  (2015) | Cotton | Alate | 86 | 84 | 0 | 2 |
|  | Cotton | Apterous | 82 | 82 | 0 | 0 |
| Oct.14  (2015) | Cotton | Alate | 94 | 94 | 0 | 0 |
|  | Cotton | Apterous | 110 | 109 | 0 | 1 |
| May.20  (2016) | Cotton | Alate | 78 | 71 | 3 | 4 |
|  | Cotton | Apterous | 65 | 60 | 4 | 1 |
